# Clinical significance of glycoprotein nonmetastatic B and its association with HER2 in breast cancer

**DOI:** 10.1002/cam4.480

**Published:** 2015-06-16

**Authors:** Masako Kanematsu, Manabu Futamura, Masafumi Takata, Siqin Gaowa, Atsuko Yamada, Kasumi Morimitsu, Akemi Morikawa, Ryutaro Mori, Hideaki Hara, Kazuhiro Yoshida

**Affiliations:** 1 Department of Surgical Oncology, Graduate School of Medicine, Gifu UniversityGifu, Japan; 2 Department of Breast and Molecular Oncology, Graduate School of Medicine, Gifu UniversityGifu, Japan; 3 Molecular Pharmacology, Department of Biofunctional Evaluation, Gifu Pharmaceutical UniversityGifu, Japan

**Keywords:** Biomarker, breast cancer, cross talk, GPNMB, HER2

## Abstract

Glycoprotein nonmetastatic B (*GPNMB*) is a potential oncogene that is particularly expressed in melanoma and breast cancer (BC). To clarify its clinical significance in BC, we measured serum GPNMB *in vivo* and investigated its cross talk with human epidermal growth factor 2 (HER2). GPNMB was expressed in four of six breast cell lines (SK-BR-3, BT-474, MDA-MD-231, and MDA-MD-157), two of six colorectal cell lines, and two of four gastric cancer (GC) cell lines. We established a GPNMB quantification system using enzyme-linked immunosorbent assay (ELISA) for these cell lines. We measured serum GPNMB in vivo in 162 consecutive BC patients and in 88 controls (50 colorectal cancer [CC] and 38 GC patients). The GPNMB concentration in BC, CC and GC was 8.163, 5.751 and 6.55 ng/mL, respectively. The GPNMB level was significantly higher in BC patients than in CC patients (*P *= 0.021). The HER2-rich subtype of BC patients had significantly higher GPNMB levels than other subtypes (vs. Luminal; *P *=* *0.038; vs. DCIS; *P *=* *0.0195). These high GPNMB levels decreased after treatment (surgery/chemotherapy). Next, we examined the relationship between GPNMB and HER2 *in vitro* using SK-BR3 and BT-474 (HER2-positive/GPNMB-positive) cells. GPNMB depletion by small interfering RNA (siRNA) increased both HER2 expression and phosphorylation. Trastuzumab (Tra) in combination with docetaxel promoted cell growth inhibition, and treatment with Tra or an Extracellular signal-related kinase (ERK) inhibitor enhanced GPNMB expression. These results indicate that GPNMB might be a surrogate marker for BC and may cross talk with the HER2 signal pathway. GPNMB may therefore emerge as an important player in anti-HER2 therapy.

## Introduction

Breast cancer (BC) is the most globally prevalent cancer that affects the female population. Despite recent progress in diagnostic and therapeutic strategies, over half a million women still die of BC annually 1. In the early 2000s, a novel molecular classification system for BC identified several BC subtypes based on gene expression patterns, including luminal A, luminal B, human epidermal growth factor receptor type 2 (HER2)-rich, normal breast-like, and basal-like 2. This striking report implied the importance of tumor biology for diagnostics and evaluation of therapeutic options. Indeed, estrogen-receptor (ER) expression is a vital indicator for endocrine therapy 3. Furthermore, advances in molecular biology have led to the development of a molecular target representative of the HER2 BC subtype. Although the prognosis for the HER2-positive primary BC patients is worse than that for HER2-negative patients, anti-HER2 therapy with drugs such as Tra, a recombinant monoclonal antibody, achieved a 44% reduction in lethality compared with HER2-negative patients 4. In addition, the extracellular domain (ECD) of HER2 has been suggested as a useful surrogate marker for HER2-positive BC 5–8.

Glycoprotein nonmetastatic B (GPNMB) is a type I transmembrane protein localized on the cell surface and in lysosomal membranes and exists in a secreted form. The GPNMB gene was originally isolated from a metastatic melanoma cell line 9. In normal tissue, this gene is also known as dendritic cell-associated heparin sulfate proteoglycan-dependent integrin ligand (DC-HIL), osteoactivin (OA), or hematopoietic growth factor inducible neurokinin-1 type (HG-FIN). It is reportedly involved in the migration of dendritic cells, in the differentiation of both osteoblasts and osteoclasts, and in the transport of melanosomes to keratinocytes 10–12. Our group previously reported that GPNMB plays an important role in neuroprotection, leading to the suppression of motor neuron degeneration such as amyotrophic lateral sclerosis (ALS) 13. Furthermore, GPNMB has been reported to be overexpressed in several cancers, including melanoma, breast, and gastric cancer 14–16. Recently emerging data has generated a more complex picture related to metastasis with respect to GPNMB in cancer progression 17–19. It was reported that the ECD of GPNMB as well as that of HER2 is cleaved by a disintegrin and metallopeptidase domain 10 (ADAM10) 20. Recently, Li et al. 21 proposed GPNMB as a prognostic indicator in small cell lung cancer. These novel discoveries prompted us to measure the serum GPNMB levels of BC patients and evaluate the clinical significance of this protein.

In this study, we describe the potential and significance of serum GPNMB level determination in BC patients. Furthermore, based on the results obtained from clinical samples, we examined the mechanism by which GPNMB contributes to tumor progression, assuming that a cross talk between GPNMB and HER2 exists through shared signaling pathways.

## Material and Methods

### Cell lines and cell culture

BC cell lines (MCF7, SK-BR-3, BT-474, MDA-MB-231, and MDA-MB-157), an immortalized normal breast cell line (MCF10), colon cancer (CC) cell lines (SW48, HCT116, LS174T, SW1417, Lovo, and Colo320DM), and GC cell lines (MKN1, MKN7, MKN45, and MKN74) used in this study were all obtained from American Type Cell Cultures (ATCC, Manassas, VA). All cell lines were cultured according to the manufacturer's guidelines.

### Reverse transcription-polymerase chain reaction

Total RNA was isolated from subconfluent cells using ISOGEN reagent (Nippon Gene, Osaka, Japan) for reverse transcription-polymerase chain reaction (RT-PCR) analysis as previously described 22. Total RNA (5 *μ*g) was reverse-transcribed with SuperScript II reverse transcriptase (Invitrogen, Carlsbad, CA). The PCR run was performed in the exponential phase (21–30 cycles) to allow semiquantitative comparisons among cDNAs synthesized from identical reactions. PCR was carried out on a Gene Amp PCR system 9700 (Takara, Otsu, Japan). The oligonucleotide primer sequences were as follows: GPNMB-forward, 5′-GGATGAGATGTGTCTGCTGAC-3′; GPNMB-reverse, 5′-GTACACCAAGAGGGAGATCA-3′; ER*α*-forward, 5′-TGGTGGAGATCTTCGACATG-3′; ER*α*-reverse, 5′-CCTGATGTGGGAGAGGATGA-3′; HER2-forward, 5′- TGTGGGCTCCCCATATGTCT-3′; HER2-reverse, 5′-CATCTGCATGGTACTCTGTCT-3′; epidermal growth factor receptor (EGFR)-forward, 5′-AAGCAACATCTCTCCGAAAGCC-3′; EGFR-reverse, 5′-GACGGTCCTCCAAGTAGTTC-3′; glyceraldehyde-3-phosphate dehydrogenase (GAPDH)-forward, 5′-CAATCACATGGTTTACATGTTC-3′; and GAPDH-reverse, 5′-GCCAGTGGATCCACGAC-3′.

### Western blotting

Cells were harvested and lysed in CelLyticTM-M (Sigma-Aldrich, St. Louis, MO) for 30 min on ice. Cell lysates (10 *μ*g/lane) were separated by SDS-PAGE using SuperSepTM (Wako, Osaka, Japan) and transferred onto Polyvinylidene difluoride (PVDF) membranes. The membranes were blocked for 1 h and then incubated with the respective primary antibody overnight at 4°C. The antibody against GPNMB was from R&D Systems (Minneapolis, MN), antibodies against HER2 and phospho-HER2 were purchased from EPITOMICS (Burlingame, CA), and all other antibodies (EGFR, phospho-EGFR, p42/44 MAPK [ERK], phospho-p42/44 Mitogen-activated protein kinase (MAPK) [ERK], and *β*-actin) were obtained from Cell Signaling Technology (Danvers, MA). The membranes were washed and incubated with the appropriate secondary antibodies. The immunoreactive proteins were visualized and captured by an LAS-4000 system (FUJIFILM, Tokyo, Japan).

### Immunocytochemistry

BC cell lines (MCF7, BT-474, SK-BR-3, and MDA-MB-157) were seeded in an eight-well chamber slide. Twenty-four hours later, the cells were fixed with 4% paraformaldehyde for 15 min, and then blocked with 3% BSA in PBS for 1 h at room temperature. The cells were incubated with the anti-GPNMB antibody (1:100) 1 h at room temperature; this was followed by incubation with goat anti-mouse secondary antibody (Alexa 467; Invitrogen). Finally, the cells were then stained with propidium iodine and visualized under a fluorescent microscope IX50 (Olympus, Tokyo, Japan) 23.

### Patients and sample collection

This study was approved by the central ethics committee of Gifu University. Between May 2011 and February 2013, 162 consecutive BC patients who received operation or chemo/endocrine therapy (primary without distant metastasis: *n* = 119; metastatic [MBC]/stage IV: *n* = 43]) were enrolled in this study. The patients who had duplicated cancers were excluded. These patients were classified into four groups according to pathological features. Of these patients, 108 were classified as Luminal BC (HER2-negative), 25 as HER2-positive (including Luminal/HER2), 20 as TN, and nine as Ductal carcinoma in situ (DCIS) 2. All patients gave informed consent to provide a surplus of serum in the central laboratory at Gifu University Hospital. During the same period, patients that were diagnosed with CC (*n* = 50, primary and without distant metastasis: *n* = 38; metastatic/stage IV: *n* = 12) and GC (*n* = 38, primary and without distant metastasis: *n* = 21, metastatic/stage IV: *n* = 17) were also enrolled in the study as controls and provided informed consent.

### GPNMB measurement

Normal breast and BC cell lines (MCF10, SK-BR-3, BT-474, MDA-MB-157, and MDA-MB-231), CC cell lines (HCT116 and LS174T), and GC cell lines (MKN1 and MKN45) were cultured in 10-cm dishes up to 50–80% confluence. The culture medium was then changed to serum-free medium and cells were cultured for additional 48–72 h. The media were collected, centrifuged at 10,000 rpm for 1 min, and then aliquoted and frozen until measurement. The serum from each patient was also stored until measurement. Stored sera and culture media were thawed on ice, diluted, and then subjected to ELISA. In brief, the serum and the culture medium were 10-fold diluted with Reagent Diluent Concentrate 2, and then reacted with human osteoactivin/GPNMB Duo set (R&D Systems) according to the manufacturer's instructions. The antibody provided in the ELISA kit was used for ELISA, western blotting, and immunocytochemistry. The results were obtained by reading the optical density at 450 nm 21.

### Immunohistochemistry

Immunohistochemical staining was performed according to standard procedures using the same antibody described above and a biotin-conjugated donkey anti-goat secondary antibody (Jackson Laboratories, West Grove, PA). Sections were developed with diaminobenzidine (DAB) staining and were counterstained with hematoxylin. The staining intensity and ranges were determined by experienced pathologists.

### siRNA for GPNMB

Both HER2- and GPNMB-positive cell lines (SK-BR-3 and BT-474) were seeded 24 h before transfection at 50–80% confluence. The cells were transiently transfected with stealth siRNA (Invitrogen) using Lipofectamine RNAiMAX (Invitrogen) at a final concentration of 5–10 nmol/L in Opti-MEM (Invitrogen). The nucleotide sequences were as follows: 5′-GGCCUGUUUGUUUCCACCAAUCAUA-3′ (sense) and 5′-UAUGAUUGGUGGAAACAA ACAGGCC-3′ (antisense) for GPNMB-si1, 5′-CCAGCCCUCGUGGGCUCAAAUAUAA-3′ (sense) and 5′-UUAUAUUUGAGCCCACGAGGGCUGG-3′ (antisense) for GPNMBsi2. Stealth siRNA negative control (control-si; Invitrogen) was used as a control.

### Drugs

Tra was supplied by Chugai Pharmaceutical (Tokyo, Japan). Docetaxel (DTX) was purchased from Sigma-Aldrich. The ERK inhibitor FR180204 was from EMD Millipore (Billerica, MA), and the v-akt murine thymoma viral oncogene homolog (Akt) inhibitor MK-2206 from Merck Oncology (Whitehouse Station, NJ).

### Cell proliferation assay

Cell proliferation was assessed by 3-(4,5-dimethylthiazol-2-yl)-5-(3-carboxymethoxyphenyl)-2-(4-sulfophenyl)-2H-tetrazolium (MTS) assay using CellTiter 96 (Promega, Fitchburg, WI). Briefly, cells were plated in 96-well plates (1250–5000 cells per well) and allowed to adhere overnight in a humidified atmosphere of 5% CO_2_. Medium was then removed and replaced by fresh culture medium containing Tra (100 *μ*g/mL) and/or DTX (0.001–0.0005 nmol/L). CellTiter 96 reagent was added to the wells after 48, 96, or 144 h, and the cells were incubated for another 1–4 h at 37°C. The absorbance at 490 nm was measured using an Envision 2104 Multilabel Reader (Perkin-Elmer, Waltham, MA).

### Statistical analysis

All statistical analyses were performed with EZR (Saitama Medical Center, Jichi Medical University, Omiya, Japan) 24. Student's *t*-test was used to compare two groups. Statistical results were considered to be significant at a *P* < 0.05.

## Results

### Expression of GPNMB in various cancer cell lines

**Figure 1:**
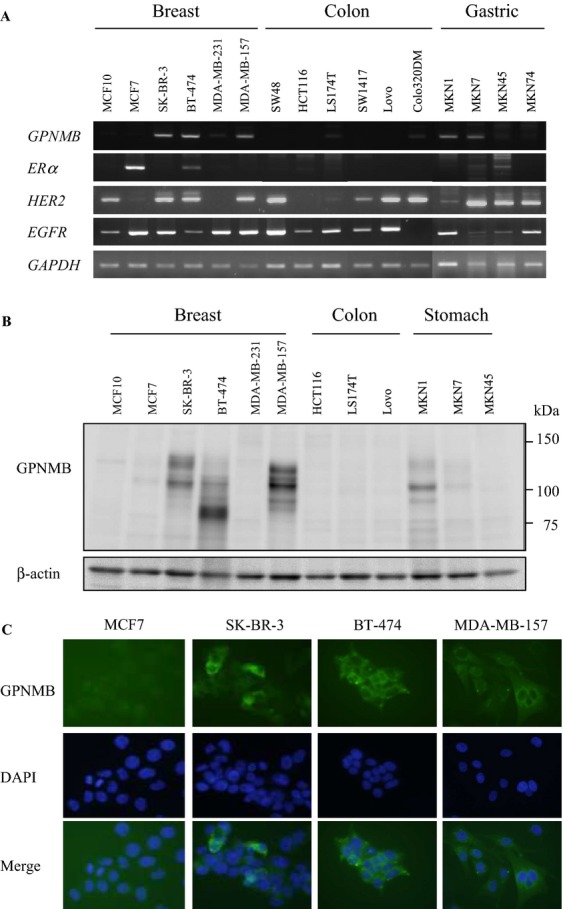
GPNMB expression in cancer cell lines. (A) Expression levels of various receptors in breast, colon, and gastric cancer cell lines assessed by RT-PCR; GAPDH was used as an internal control. (B) Expression levels of GPNMB in several cancer cell lines analyzed by western blot; *β*-actin was used as an internal control. (C) Immunocytochemistry for GPNMB with fluorescent microscopy (400×). Four BC cell lines (MCF7, BT474, SK-BR-3, and MDA-MB-157) were fixed and stained with anti-GPNMB (upper), counterstained with DAPI (middle), merged (lower), and visualized using a fluorescent microscope.

First, the expression levels of *GPNMB* were evaluated using RT-PCR and western blot analysis. All cell lines (five BC cell lines, one immortalized normal breast cell line, six CC cell lines, and four GC cell lines) were subjected to RT-PCR. *GPNMB* was expressed in four of six (67%) BC cell lines (SK-BR-3, BT-474, MDA-MB-231, and MDA-MD-157). In particular, the expression levels of three cell lines (SK-BR-3, BT-474, and MDA-MB-157) were much higher than those of the other cell lines. Although very low expression was observed in only two (LS174T and Colo320DM) of the six CC cell lines tested, two (MKN1 and MKN7) of the four GC cell lines expressed *GPNMB*. We also examined the gene expression of various receptors, including *ERα, HER2,* and *EGFR*; however, *GPNMB* expression was independent from that of other receptors related to BC (Fig.1A). Real-time quantitative RT-PCR also showed similar results to conventional RT-PCR. Three representative BC cell lines (SK-BR-3, BT-474, and MDA-MB-157) also showed high expression levels of *GPNMB* (Fig. S1).

We confirmed the expression of GPNMB by western blot. The expression levels and patterns of endogenous GPNMB observed were consistent with those detected by RT-PCR. In particular, SK-BR-3, BT-474, MDA-MB-157, and MKN1 cells expressed a sufficient amount of GPNMB to be detectable by western blot. Differences in the band size seemed to be derived from splice variants or protein modifications such as glycosylation (Fig.1B). We further confirmed GPNMB expression by immunocytochemistry using four BC cell lines. As indicated by RT-PCR and western blotting, a strong GPNMB expression was detected in SK-BR-3, BT474, and MDA-MB-157 cells, but the expression was weak in MCF7 cells (Fig.1C). As reported previously, GPNMB was localized intracellularly both to the cell membrane and the cytoplasm, particularly in the peri-nuclear area 10,25.

### Measurement of GPNMB in cell culture media

**Figure 2:**
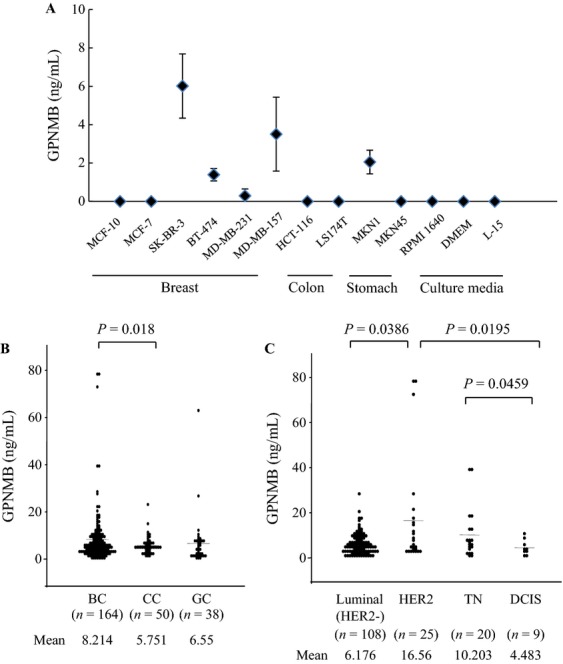
GPNMB measurement in cell culture media and in vivo. (A) Cell culture media were subjected to GPNMB measurement. Three culture media (RPMI1640, DMEM, and L-15) were used as controls. Error bars indicate standard deviations. (B) GPNMBs in patients with breast (BC: *n* = 164), colon (CC: *n* = 50), or gastric (GC: *n* = 38) cancer are indicated, respectively. (C) GPNMBs in four subgroups of BC patients (Luminal: *n* = 108, HER2-positive: *n* = 25, TN: *n* = 20, and DCIS: *n* = 9) are shown, respectively.

In addition to the potential of GPNMB as an oncogene, some proteases representative of ADAM10 have been reported to shed the ECD of GPNMB 21. Therefore, we speculated that the soluble shedding of GPNMB in serum might be measurable and could be associated with BC progression. As a preliminary analysis, we measured and evaluated the soluble GPNMB levels in culture media from various cell lines incubated for 48–72 h (>80% confluence) by ELISA. Interestingly, we found that culture media from the SK-BR-3, BT474, MDA-MB-157, and MKN1 cell lines contained apparent soluble GPNMB (Fig.2A). These results were consistent with the results obtained from RT-PCR and western blot analysis, suggesting that shed GPNMB might be detectable even in sera obtained from BC or GC patients with high GPNMB expression.

### Measurement of GPNMB in vivo

In order to measure the shed GPNMB *in vivo*, we collected and analyzed a total of 250 serum samples from BC (*n* = 162), CC (*n* = 50), and GC (*n* = 38) patients. The mean GPNMB concentrations in the BC, CC, and GC groups were 8.163 (±11.293) ng/mL, 5.751 (±3.764) ng/mL, and 6.55 (±10.541) ng/mL, respectively; the BC group showed significantly higher levels of serum GPNMB than the CC groups (*P *=* *0.021) (Fig.2B). Within the BC group, the mean GPNMB concentration was 8.079 (±11.348) ng/mL in patients with local disease (stages 0, I, II, or III) and 8.394 (±11.308) ng/mL in patients with stage IV or MBC; however, there was no significant difference between patients with local disease and MBC. With respect to the BC subtypes, the shed GPNMB level of the HER2-positive group was significantly higher than that of the other subtype and DCIS (comparison with Luminal group; *P *=* *0.0386, DCIS; *P *=* *0.0195). Furthermore, the GPNMB levels of the TNBC group were also higher than those of the DCIS group (*P *=* *0.0459), as shown in Figure2C. There were at least 11 patients with >15 ng/mL of GPNMB, including nine (8.3%) BC patients (two Luminal, five HER2-positive, and two TNBC cases), one (2%) CC patient (stage II), and one (3.2%) GC patient (stage I). To confirm GPNMB expression in tumor cells, we also performed immunohistochemistry using 10 surgical/biopsy specimens for the HER2-positive group. Of six HER2-positive BC patients with high GPNMB levels (>15 ng/mL), five samples showed GPNMB expression in tumor cells. In contrast, three of four HER2-rich BC patients with low GPNMB (<5 ng/mL) showed faint or negative staining in tumor cells irrespective of a positive signal in stroma (Fig. S2).

### Potential of GPNMB as a predictive factor

**Figure 3:**
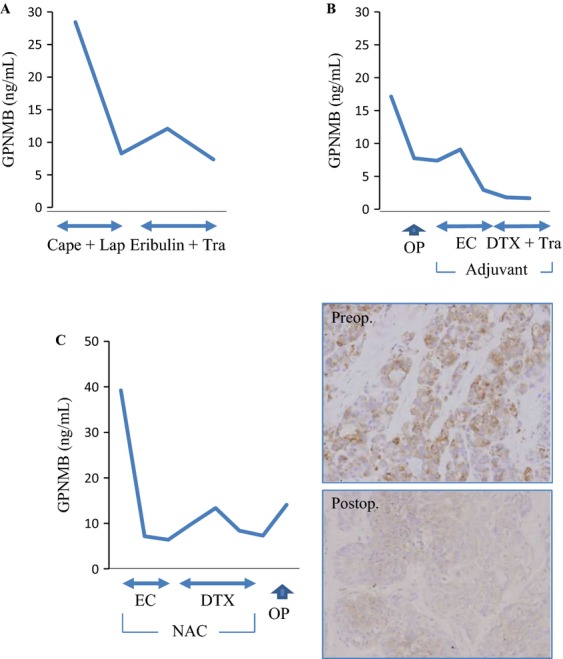
Changes in serum GPNMB levels after treatments. Sequential GPNMB levels and therapeutic processes are indicated for three representative cases. (A) A 66-year-old woman with metastatic HER2-rich BC was treated with capecitabine (Cape) and lapatinib (Lap) followed by eribulin plus trastuzumab (Tra). (B) A 68-year-old woman with Luminal/HER2 BC (stage IIB) was treated with mastectomy. EC followed by DTX and Tra was performed as an adjuvant therapy. (C) A 60-year-old woman with TNBC (stage IIB) had EC followed by DTX as neoadjuvant chemotherapy (NAC), then a mastectomy was performed. GPNMB staining is shown using tumor specimens before and after chemotherapy (200×).

In this study, we detected some patients with high serum GPMNB levels, particularly in the BC group. In order to confirm the potential of GPNMB as a surrogate marker for treatment, we also monitored the sequential GPNMB levels throughout therapeutic procedures. We here describe the results of three representative cases. First, a 66-year-old woman suffering from metastatic HER2-positive BC was treated with capecitabine (Cape) (Chugai, Tokyo, Japan) and lapatinib (Lap) (Glaxo, Middlesex, UK) followed by eribulin (Eizai, Tokyo, Japan) plus Tra. Her disease remained clinically stable and her GPNMB levels decreased from 28.43 to 7.37 ng/mL within 8 months (Fig.3A). Second, a 68-year-old woman with Luminal/HER2 BC (stage IIB) was treated with mastectomy followed by sequential chemotherapy including EC (epirubicin and cyclophosphamide) and then DTX and Tra as adjuvant therapy. Her GPNMB levels decreased from 17.15 to 1.67 ng/mL within 12 months without relapse (Fig.3B). Third, a 60-year-old woman with TNBC (stage IIB) had neoadjuvant chemotherapy (NAC) (EC followed by DTX) followed by mastectomy. Although the disease remained clinically stable, her GPMNB level decreased by chemotherapy. To evaluate the expression of GPNMB in relation to the pathological status, we compared GPNMB levels in preoperative and postoperative specimens. Despite no apparent shrinkage by radiological evaluation, some part of the remnant tumor tissue had become necrotic. As expected, GPNMB staining of the viable area of the postoperative sample (excluding the necrotic tissue) was clearly weaker than that of the preoperative sample (Fig.3C). During periods of low GPNMB levels, each patient's disease was stably controlled. These results suggest that serum GPNMB might be useful for monitoring BC patients that overexpress GPNMB.

### Relationship between GPNMB and HER2

**Figure 4:**
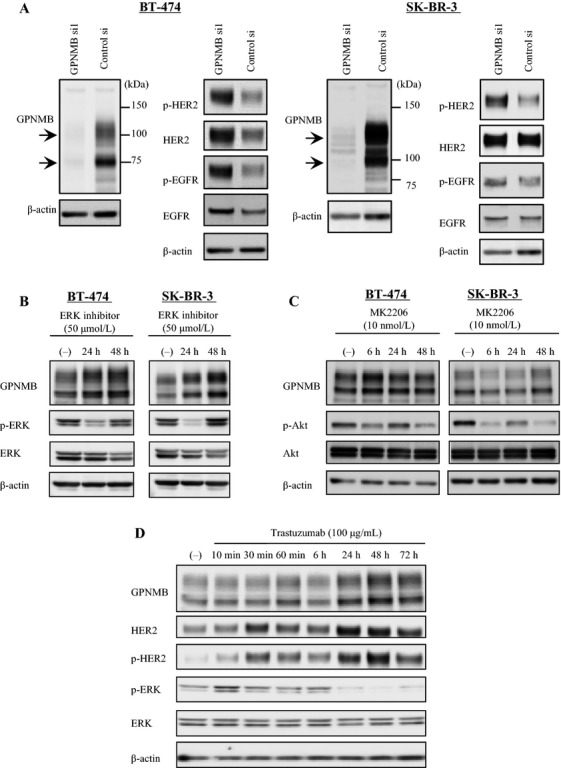
Association between GPNMB and membrane receptor families, including HER2 and EGFR. (A) GPNMB-targeted siRNA was transiently transfected into BT-474 (right) and SK-BR-3 (left) cells. GPNMB expression (arrows) and concomitant increases in HER2 and EGFR expression and their phosphorylated forms are indicated. Control-si was used as a negative control. (B) GPNMB expression changes in BT-474 and SK-BR-3 cells after ERK inhibition. (C) GPNMB expression change in BT-474 and SK-BR-3 cells after Akt inhibition. (D) Increased GPNMB expression in BT-474 cells after Tra treatment.

On the basis of the data of serum GPNMB *in vivo*, we paid much attention to a possible relationship between GPNMB and growth factor receptors, particularly HER2. At first we investigated knockdown of GPNMB using SK-BR-3 and BT-474 cells that express high levels of GPNMB as shown in Figure1. GPNMB-specific siRNAs were transfected into these cells, and the expression levels of GPNMB and certain growth factors were evaluated by western blot 48 h later. The expression of HER2 and EGFR increased in BT-474 cells after GPNMB depletion. Furthermore, the phosphorylation of HER2 was apparently elevated in both cells, suggesting that the signaling pathway of GPNMB may be cross-linked with that of the EGFR receptor families such as HER2 and/or EGFR (Fig.4A). In order to confirm this phenomenon, we observed GPNMB expression after inhibition of ERK and Akt, which act as the main downstream pathways for both HER2 and EGFR. As shown in Figure4B and C, GPNMB expression increased after ERK inhibition in both cell lines. However, Akt inhibition contributed little to GPNMB expression.

Next, we investigated a potential expression change in GPNMB after inhibition of HER2 by Tra, which is a HER2-targeted monoclonal antibody. Twenty-four hours after treating BT-474 cells with Tra (100 *μ*g/mL), ERK phosphorylation was almost completely inhibited. In contrast, the treatment increased GPNMB expression as well as HER2 expression and phosphorylation (Fig.4D). These results suggest that there might be a strong cross talk between GPNMB and HER2.

### Effect of GPNMB depletion on cell growth

**Figure 5:**
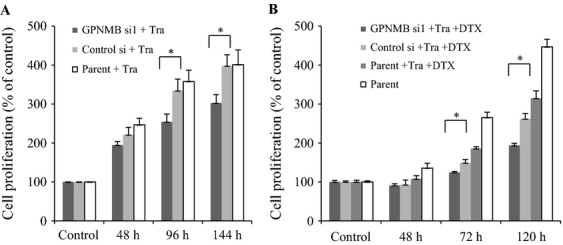
Effect of GPNMB depletion on treatment with Tra and/or DTX in SK-BR-3 cells. (A) Three kinds of SK-BR-3 cells (GPNMB-si1 transfected, control-si transfected, and parent cells) were treated with Tra and the cell growth was compared (*P <* *0.0001). (B) Cells were treated with both Tra (100 *μ*g/mL) and DTX (0.0005 nmol/L) and the cell growth was evaluated (*P <* *0.0001).

On the basis of the results described above, we assumed that GPNMB may influence anti-HER2 therapy if there is a cross talk between GPNMB and HER2. In order to confirm this speculation, we investigated the cell growth after knocking down GPNMB. First, we transfected SK-BR-3 (HER2-positive) cells with siRNA for GPNMB (GPNMB-si1) and control-si, and then treated the cells with Tra. Interestingly, cell proliferation of GPNMB-depleted SK-BR-3 cells was significantly suppressed following Tra treatment compared with that of the control (Fig.5A) (*P *<* *0.0001). Because Tra shows a strong anti-cancerous effect in combination with anticancer drugs in vivo and in vitro 26, we further treated GPNMB-depleted cells with both Tra and DTX, a representative anti-BC drug. Interestingly, the combination of Tra with DTX enhanced the cell growth suppression beyond that obtained with Tra monotherapy (Fig.5B) (*P *<* *0.0001). A similar phenomenon was also observed when another siRNA (GPNMB-si2) (was used (Fig. S3). These results strongly indicated that GPNMB may be associated with the HER2 signaling pathway and could play an important role in anti-HER2 therapy.

## Discussion

The role of GPNMB in cancer was first discussed based on expression analyses demonstrating that GPNMB expression is particularly high in melanoma and BC cell lines in vitro 14. Emerging expression analyses using clinical samples also suggest GPNMB as a potential oncogene in several cancers, including melanoma, glioma, breast, and GC 14–19. With regard to BC, Rose et al. 15 demonstrated that GPNMB is expressed in highly proliferative BCs such as Luminal B, HER2-rich, and basal-like BC and is associated with the prognosis. As ECD of GPNMB is shed and released like HER2, we speculated that shed GPNMB would be measurable and could serve as a useful surrogate marker. On the basis of this idea, we investigated the clinical significance by measuring serum GPNMB in patients with BC.

Similar to *in vitro* expression analysis, the GPNMB means were significantly higher in BC patients than in CC patients, which is in agreement with the previous data 14. Among BC patients, GPNMB in the HER2 type was significantly higher than in the Luminal or DCIS group, indicating that GPNMB might play a crucial role in HER2-positive BC. Nevertheless, GPNMB in MBC was higher than in operable cases (8.341 and 8.15 ng/mL, respectively), with no statistical difference. The source of shed GPNMB needs to be determined, because it may originate not only from cancer cells but also from noncancerous tissue such as bone, thymus, and adipose tissue 27. Even within BC tissues, there are two expression patterns for GPNMB, namely the epithelial type and the stromal type; at present, our system does not strictly segregate the precise origins of serum GPNMB. We also measured GPNMB for 16 patients with benign breast disease, including patients with fibroadenoma and mastopathy, and cancer-free patients, whose GPNMB level was 8.029 ng/mL which was not statistically different from that of BC, CC, and GC patients. The GPNMB levels varied from 0.466 to 44.435. In addition, patients with benign diseases, such as cholecystitis, or (seemingly) healthy volunteers showed high levels of GPNMB without malignancy. In addition, the high rate of Luminal type with low GPNMB in the MBC/Stage IV population (67%) (perhaps low proliferative MBC) may affect the results. On the other hand, we confirmed that both the serum GPNMB and GPNMB expression *in vivo* decreased by the employed treatments, indicating that high serum GPNMB could be used as a potential surrogate marker in some populations (Fig.3). Li et al. 21 recently reported that GPNMB is a prognostic factor for non-small cell lung cancer based on ELISA. A direct comparison between these findings and our results is difficult, because GPNMB expression is as low in lung cancer cells as it is in colon, ovary, and renal cancer cells 10,11,14, and a different measuring system was employed in their study. However, these observations suggest GPNMB as a potential tumor/surrogate marker.

The present clinical study suggested an association of GPNMB with other growth factors, particularly HER2. It is well known that a signal cross talk between ER and growth factors exists in BC, which may present one of the mechanisms for resistance against either endocrine or anti-HER2 therapy 28,29. For example, with respect to endocrine therapy, acquired resistance to tamoxifen is associated with decreased ER positivity and increased HER2 expression 28. In ER-positive BC cells that have developed endocrine resistance, ER expression may be suppressed directly by enhanced peptide growth factor receptor signaling that is due to overexpression of the type I growth factor receptors, such as EGFR and HER2, with subsequent downstream MAPK activation that inhibits ER transcriptional expression 29. We now recognize that biomarkers or therapeutic targets such as ER and HER2 may not necessarily remain constant, but change through interaction with each other. The detection of novel biomarkers, especially those with a correlation with representative therapeutic targets processing to treatment tolerance, will advance the development of improved BC treatment. Our data demonstrate that GPNMB depletion induced not only the expression of HER2 and EGFR but also their phosphorylation. On the other hand, blockage of HER2 function by Tra also induced GPNMB expression with a concomitant decrease in phosphorylated ERK, strongly suggesting that GPNMB may be linked to HER2 through intracellular signaling pathways and particularly the MAPK pathway. Nonetheless, its mechanisms of action remain controversial and poorly understood. Gijsen et al. 30 reported the upregulation and phosphorylation of HER1/2/3/4 after Tra administration in HER2-overexpressing cells and maintenance of HER2 phosphorylation by ligand-mediated activation of HER1/3/4. These phenomena are strongly associated with increased activation of MAPK pathways, and the authors concluded that these factors may be involved in the resistance to anti-HER2 therapy. GPNMB is assumed to be a membrane receptor, but its ligands and downstream signaling pathways remain unknown. Qian et al. 14 reported that exposure of melanoma cells expressing high levels of GPNMB to an ERK inhibitor strongly induced GPNMB. We obtained similar results in BC cells with high GPNMB expression. In contrast, we did not observe induction of GPNMB expression in the LS174T CC cell line (data not shown). These results suggest that inhibition of the MAPK pathway representative of ERK is involved in GPNMB signaling in a tumor-specific manner. Detailed analysis of this intriguing GPNMB signaling pathway should be further investigated.

**Figure 6:**
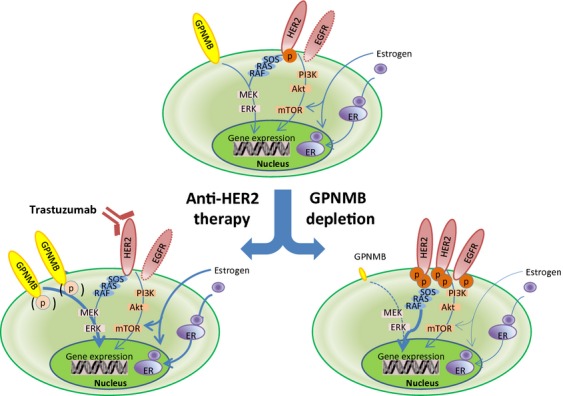
Putative intracellular association of GPNMB. In HER2-positive cells, GPNMB may crosstalk with membrane receptors. GPNMB may be induced in accordance with anti-HER2 therapy. When GPNMB is depleted, growth factors such as HER2 and EGFR may be activated.

We further aimed to delineate the signaling cross talk between GPNMB and HER2. We could demonstrate that combined GPNMB depletion and Tra treatment led induced a stronger effect on HER2-positive tumor suppression than Tra treatment alone. Additional chemotherapy (DTX) further enhanced tumor suppression in vitro. Recent studies have shown that Tra induces HER2 receptor down-regulation, but does not decrease HER2 phosphorylation or HER2 activation 31–33. This may be the reason why patients invariably develop resistance if they receive Tra monotherapy. So far, major molecular mechanisms of Tra resistance may involve signaling from other growth factor receptors, including HER3, insulin-like growth factor receptor1 (IRF-R1), c-MET, or loss of phosphatase and TENsin homolog (PTEN), and expression of the p95 isoform of HER2 34. Although a decade has passed since anti-HER2 therapy (Tra) has been approved for clinical use, resistance to anti-HER2 therapy persists, despite the discovery of other anti-HER2 therapeutic agents, including pertuzumab for inhibition of the HER2-HER3 heterodimer and Lap for the inhibition of intracellular tyrosine kinase for HER1/HER2 35,36. GPNMB could potentially participate in these pathways and may play an important role in signal transduction in BC (Fig.6).

A GPNMB-targeted antibody drug conjugate called CR011-maleimidocaproyl-valinecitruline-monomethyl auristatin E (CR011-vcMMAE) has been proven experimentally useful for GPNMB-expressing tumors *in vitro*. Phase I/II studies using CR011-vcMMAE called for glembatumumab vedotin (Celldex therapeutics, Canton, MA) for MBC and advanced melanoma have been performed in the United States 27,37. Very recently, the results of phase I trials have been reported that glembatumumab vedotin is clinically useful for TNBC or high GPNMB-positive tumors with longer progression-free survival (PFS) than GPNMB-negative tumors 27. These positive results were also observed in metastatic melanoma patients with 33% of objective response rate (ORR) 37. These reports indicate that GPNMB may be clinically important and may serve as a promising therapeutic target. Our findings indicate that glembatumumab vedotin may be used to treat HER2-positive MBC. Large-scale cohort studies are needed to further clarify the significance of GPNMB in BC.

## Conclusion

We discovered that serum GPNMB levels were high in BC patients, particularly in those with the HER2-rich subtype, which is a potential surrogate BC marker. Furthermore, GPNMB may cross talk with HER2 signaling pathways and play an important role in anti-HER2 therapy.

## Supporting Information

**Data S1.** Supplementary materials and methods.

Figure S1. GPNMB expression by real-time quantitative (RT-PCR). cDNAs similar to Figure 1A were used for this analysis. Each expression levels was compared with the minimum expression level (Lovo cells), and the relative quantification (RQ) was determined. **Figure S2.** Immunohistochemistry of breast cancer samples of the HER2-rich subtype. Representative cases of immunohistochemistry were indicated with the serum GPNMB level. **Figure S3.** Effect of GPNMB depletion on treatment with Tra and/or DTX in SK-BR-3 cells. GPNMB si2 was transfected to SK-BR-3 cells, and then treated with either Tra or DTX. (A) SK-BR-3 cells (GPNMB-si2 transfected and control-si transfected) were treated with Tra (100 *μ*g/mL) and cell growth was compared (**P *<* *0.00001). (B) Cells were treated with both Tra (100 *μ*g/mL) and DTX (0.001 nmol/L) and cell growth was evaluated (**P *<* *0.00001).

## References

[b1] Jemal A, Siegel R, Ward E, Hao Y, Xu J, Murray T (2008). Cancer statistics. CA Cancer J. Clin.

[b2] Perou CM, Sørlie T, Eisen MB, van de Rijn M, Jeffrey SS, Rees CA (2000). Molecular portraits of human breast tumors. Nature.

[b3] Bartlett JM, Brookes CL, Robson T, van de Velde CJ, Billingham LJ, Campbell FM (2011). Estrogen receptor and progesterone receptor as predictive biomarkers of response to endocrine therapy: a prospectively powered pathology study in the tamoxifen and exemestane adjuvant multinational trial. J. Clin. Oncol.

[b4] Dawood S, Broglio K, Buzdar AU, Hortobagyi GN, Giordano SH (2009). Prognosis of women with metastatic breast cancer by HER2 status and trastuzumab Treatment: an institutional-based review. J. Clin. Oncol.

[b5] Köstler WJ, Schwab B, Singer CF, Neumann R, Rücklinger E, Brodowicz T (2004). Monitoring of serum Her-2/neu predicts response and progression-free survival to trastuzumab-based treatment in patients with metastatic breast cancer. Clin. Cancer Res.

[b6] Lüftner D, Cheli C, Mickelson K, Sampson E, Possinger K (2004). ADVIA Centaur HER-2/neu shows value in monitoring patients with metastatic breast cancer. Int. J. Biol. Markers.

[b7] Ludovini V, Gori S, Colozza M, Pistola L, Rulli E, Floriani I (2008). Evaluation of serum HER2 extracellular domain in early breast cancer patients: correlation with clinicopathological parameters and survival. Ann. Oncol.

[b8] Hayashi N, Nakamura S, Tokuda Y, Yagata H, Yoshida A, Ota H (2012). Serum HER2 levels determined by two methods in patients with metastatic breast cancer. Int. J. Clin. Oncol.

[b9] Weterman MA, Ajubi N, van Dinter IM, Degen WG, van Muijen GN, Ruitter DJ (1995). nmb, a novel gene, is expressed in low-metastatic human melanoma cell lines and xenografts. Int. J. Cancer.

[b10] Shikano S, Bonkobara M, Zukas PK, Ariizumi K (2001). Molecular cloning of a dendritic cell-associated transmembrane protein, DC-HIL, that promotes RGD-dependent adhesion of endothelial cells through recognition of heparan sulfate proteoglycans. J. Biol. Chem.

[b11] Safadi FF, Xu J, Smock SL, Rico MC, Owen TA, Popoff SN (2001). Cloning and characterization of osteoactivin, a novel cDNA expressed in osteoblasts. J. Cell. Biochem.

[b12] Bandari PS, Qian J, Yehia G, Joshi DD, Maloof PB, Potian J (2003). Hematopoietic growth factor inducible neurokinin-1 type: a transmembrane protein that is similar to neurokinin 1 interacts with substance P. Requl. Pept.

[b13] Tanaka H, Shimazawa M, Kimura M, Takata M, Tsuruma K, Yamada M (2012). The potential of GPNMB as novel neuroprotective factor in amyotrophic lateral sclerosis. Sci. Rep.

[b14] Qian X, Mills E, Torgov M, LaRochelle WJ, Jeffers M (2008). Pharmacologically enhanced expression of GPNMB increases the sensitivity of melanoma cells to the CR011-vcMMAE antibody-drug conjugate. Mol. Oncol.

[b15] Rose AA, Grosset AA, Dong Z, Russo C, Macdonald PA, Bertos NR (2010). Glycoprotein nonmetastatic B is an independent prognostic indicator of recurrence and a novel therapeutic target in breast cancer. Clin. Cancer Res.

[b16] Rho HW, Lee BC, Choi ES, Choi IIJ, Lee YS, Goh SH (2010). Identification of valid reference genes for gene expression studies of human stomach cancer by reverse transcription-qPCR. BMC Cancer.

[b17] Rose AA, Pepin F, Russo C, Abou Khalil JE, Hallett M, Siegel PM (2007). Osteoactivin promotes breast cancer metastasis to bone. Mol. Cancer Res.

[b18] Kuan CT, Wakiya K, Dowell JM, Herndon JE, Reardon DA, Graner MW (2006). Glycoprotein nonmetastatic melanoma protein B, a potential molecular therapeutic target in patients with glioblastoma multiforme. Clin. Cancer Res.

[b19] Maric G, Rose AA, Annis MG, Siegel PM (2013). Glycoprotein non-metastatic b (GPNMB): a metastatic mediator and emerging therapeutic target in cancer. Onco Targets Ther.

[b20] Rose AA, Annis MG, Dong Z, Pepin F, Hallett M, Park M (2010). ADAM10 releases a soluble form of the GPNMB/Osteoactivin extracellular domain with angiogenic properties. PLoS One.

[b21] Li YN, Zhang L, Li XL, Cui DJ, Zheng HD, Yang SY (2014). Glycoprotein nonmetastatic B as a prognostic indicator in small cell lung cancer. APMIS.

[b22] Futamura M, Kamino H, Miyamoto Y, Kitamura N, Nakamura Y, Ohnishi S (2007). Possible role of semaphorin 3F, a candidate tumor suppressor gene at 3p21.3, in p53-regulated tumor angiogenesis suppression. Cancer Res.

[b23] Ohnishi S, Futamura M, Kamino H, Nakamura Y, Kitamura N, Miyamoto Y (2010). Identification of NEEP21, encoding neuron-enriched endosomal protein of 21 kDa, as a transcriptional target of tumor suppressor p53. Int. J. Oncol.

[b24] Kanda Y (2013). Investigation of the freely available easy-to-use software “EZR” for medical statistics. Bone Marrow Transplant.

[b25] Abdelmagid SM, Barbe MF, Rico MC, Salihoglu S, Arango-Hisijara I, Selim AH (2008). Osteoactivin, an anabolic factor that regulates osteoblast differentiation and function. Exp. Cell Res.

[b26] Pegram MD, Konecny GE, O'Callaghan C, Beryt M, Pietras R, Slamon DJ (2004). Rational combinations of trastuzumab with chemotherapeutic drugs used in the treatment of breast cancer. J. Natl. Cancer Inst.

[b27] Bendell J, Saleh M, Rose AA, Siegel PM, Hart L, Sirpal S (2014). Phase I/II study of the antibody-drug conjugate glembatumumab vedotin in patients with locally advanced or metastatic breast cancer. J. Clin. Oncol.

[b28] Dowsett M, Johnston S, Martin LA, Salter J, Hills M, Detre S (2005). Growth factor signalling and response to endocrine therapy: the Royal Marsden Experience. Endocr. Relat. Cancer.

[b29] Johnston SR (2010). New strategies in estrogen receptor-positive breast cancer. Clin. Cancer Res.

[b30] Gijsen M, King P, Perera T, Parker PJ, Harris AL, Larijani B (2010). HER2 phosphorylation is maintained by a PKB negative feedback loop in response to anti-HER2 Herceptin in breast cancer. PLoS One.

[b31] Scaltriti M, Verma C, Guzman M, Jimenez J, Parra JL, Pedersen K (2009). Lapatinib, a HER2 tyrosine kinase inhibitor, induces stabilization and accumulation of HER2 and potentiates trastuzumab-dependent cell cytotoxicity. Oncogene.

[b32] Junttila TT, Akita RW, Parsons K, Fields C, Lewis Phillips GD, Friedman LS (2009). Ligand-independent HER2/HER3/PI3K complex is disrupted by trastuzumab and is effectively inhibited by the PI3K inhibitor GDC-0941. Cancer Cell.

[b33] Gijsen M, King P, Perera T, Parker PJ, Harris AL, Larijani B (2010). HER2 phosphorylation is maintained by a PKB negative feedback loop in response to anti-HER2 herceptin in breast cancer. PLoS Biol.

[b34] Lavaud P, Andre F (2014). Strategies to overcome trastuzumab resistance in HER2-overexpressing breast cancers: focus on new data from clinical trials. BMC Med.

[b35] Cameron D, Casey M, Oliva C, Newstat B, Imwalle B, Geyer CE (2010). Lapatinib plus capecitabine in women with HER-2-positive advanced breast cancer: final survival analysis of a phase III randomized trial. Oncologist.

[b36] Baselga J, Cortés J, Kim SB, Im SA, Hegg R, Im YH (2012). Pertuzumab plus trastuzumab plus docetaxel for metastatic breast cancer. N. Engl. J. Med.

[b37] Ott PA, Hamid O, Pavlick AC, Kluger H, Kim KB, Boasberg PD (2014). Phase I/II study of the antibody-drug conjugate glembatumumab vedotin in patients with advanced melanoma. J. Clin. Oncol.

