# Supplementary material for: Clinical significance of glycoprotein nonmetastatic B and its association with HER2 in breast cancer
Source: Cancer Med. 2015 Jun 16;4(9):1344–55. doi: 10.1002/cam4.480 (PMC4567019; doi:10.1002/cam4.480)
Supplement: Supplementary file 1 — Data S1. Supplementary materials and methods. [file cam40004-1344-sd1.docx]

Supplementary methods

Real-time quantitative reverse transcription-polymerase chain reaction (RT-PCR)

These assays were performed according to the manufacturer’s instructions (Life technologies, Carlsbad, CA, USA). In brief, the templates described above were used for real-time quantitative RT-PCR (95 °C for 10 min for 1 cycle, 95 °C for 15 sec and 60 °C for 1 min for 40 cycles) employing TaqMan® Gene Expression Assay (Life technologies) and TaqMan® Universal Master Mix II, no UNG. Primer pairs and TaqMan probes were designed by Life technologies. Each sample was analyzed in triplicate.
