# Supplementary figures and images for: Clinical significance of glycoprotein nonmetastatic B and its association with HER2 in breast cancer
Source: Cancer Med. 2015 Jun 16;4(9):1344–55. doi: 10.1002/cam4.480 (PMC4567019; doi:10.1002/cam4.480)

Supplementary figure 1

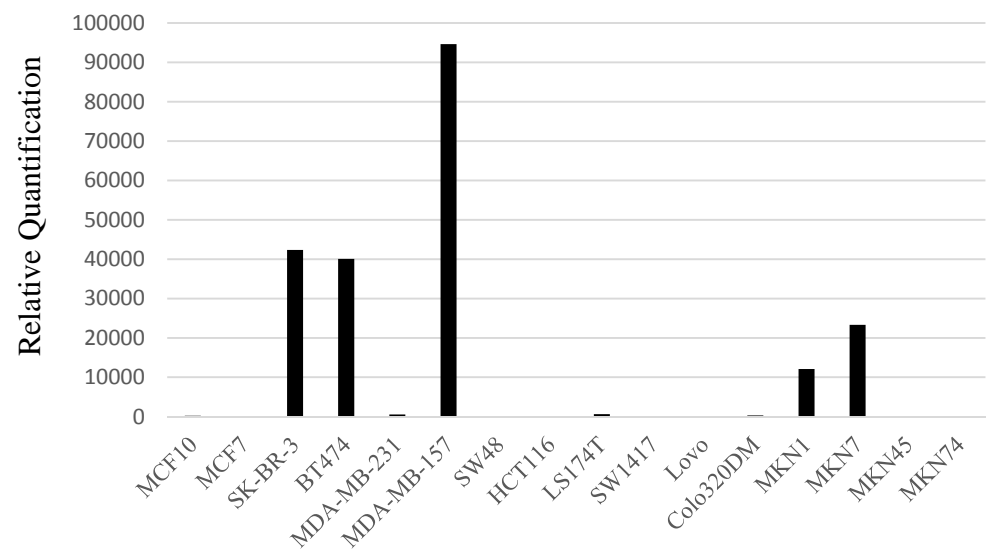

Supplementary figure 2

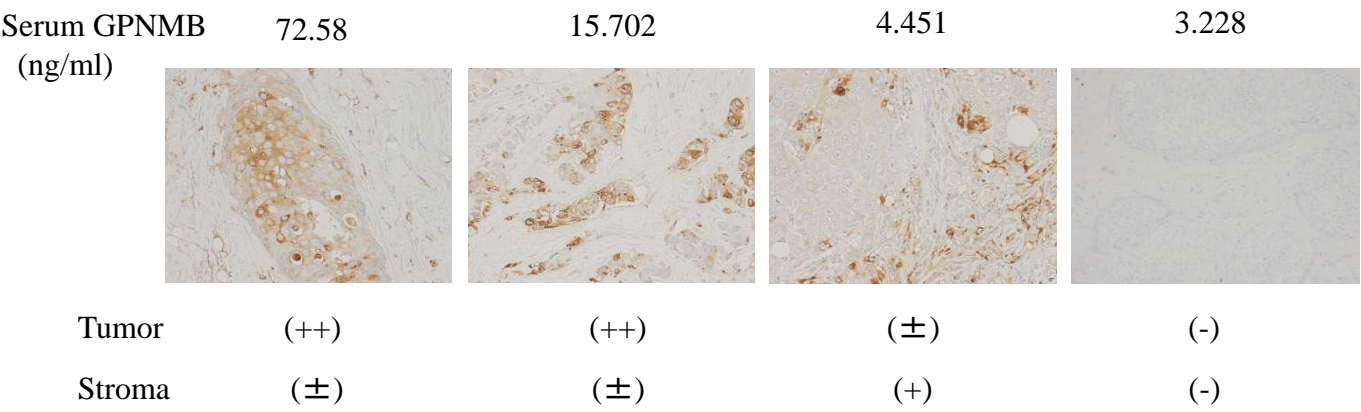

## Supplementary figure 3

**a**

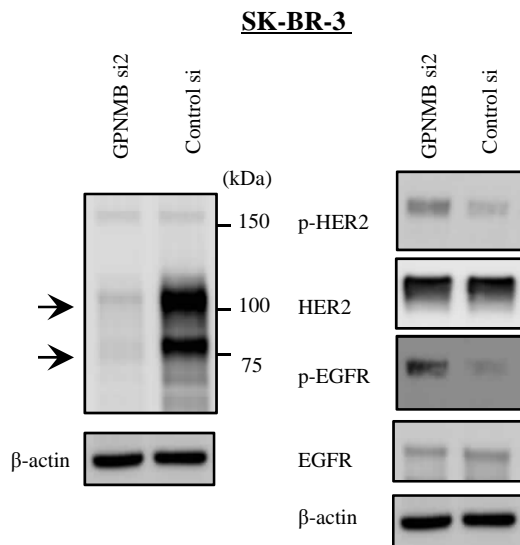

**b**

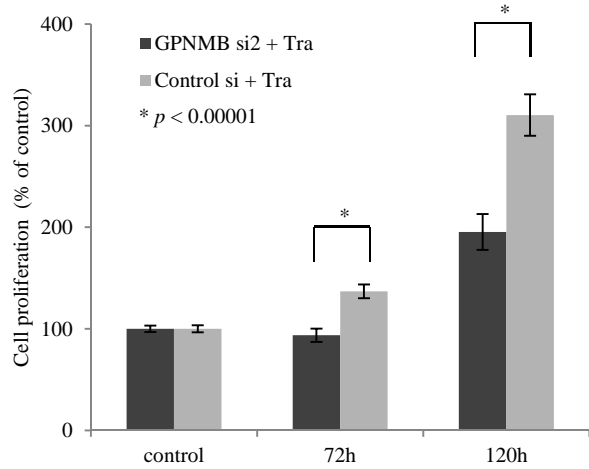

**c**

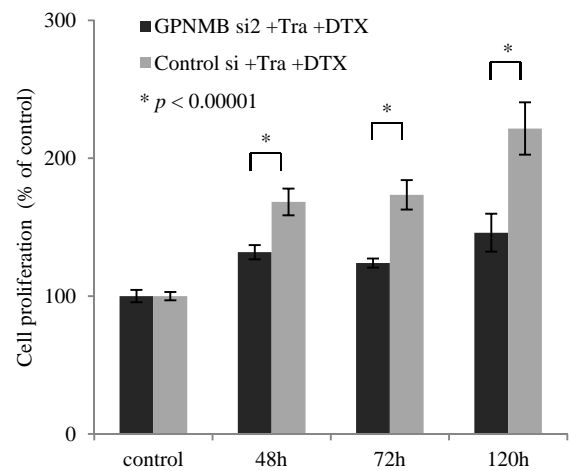

Supplement: Figure S1. — GPNMB expression by real-time quantitative (RT-PCR). cDNAs similar to Figure 1A were used for this analysis. Each expression levels was compared with the minimum expression level (Lovo cells), and the relative quantification (RQ) was determined. Figure S2. Immunohistochemistry of breast cancer samples of the HER2-rich subtype. Representative cases of immunohistochemistry were indicated with the serum GPNMB level. Figure S3. Effect of GPNMB depletion on treatment with Tra and/or DTX in SK-BR-3 cells. GPNMB si2 was transfected to SK-BR-3 cells, and then treated with either Tra or DTX. (A) SK-BR-3 cells (GPNMB-si2 transfected and control-si transfected) were treated with Tra (100 μg/mL) and cell growth was compared (*P < 0.00001). (B) Cells were treated with both Tra (100 μg/mL) and DTX (0.001 nmol/L) and cell growth was evaluated (*P < 0.00001). [file cam40004-1344-sd2.pdf]
